# The Effect of the COVID-19 Pandemic on Non–COVID-19 Deaths: Population-Wide Retrospective Cohort Study

**DOI:** 10.2196/41792

**Published:** 2024-02-13

**Authors:** Abraham Ka-chung Wai, Tsz Fung Yip, Yui Hang Wong, Chun Kit Chu, Teddy Lee, Ken Hung On Yu, Wang Leong So, Janet Y H Wong, Carlos King-ho Wong, Joshua W Ho, Timothy Rainer

**Affiliations:** 1 Department of Emergency Medicine The University of Hong Kong Hong Kong China (Hong Kong); 2 Department of Accident & Emergency The University of Hong Kong–Shenzhen Hospital Shenzhen China; 3 Department of Accident & Emergency Queen Mary Hospital Hong Kong China (Hong Kong); 4 School of Biomedical Sciences The University of Hong Kong Hong Kong China (Hong Kong); 5 School of Nursing & Health Studies Hong Kong Metropolitan University Kowloon China (Hong Kong); 6 Department of Family Medicine and Primary Care The University of Hong Kong Hong Kong China (Hong Kong)

**Keywords:** excess death, causal inference, health care avoidance, emergency department, COVID-19, hospital avoidance behavior, mortality, epidemiology, health care, hospital care, death rate, death, hospital, avoidance, population, cohort, death toll, impact, excess, Hong Kong

## Abstract

**Background:**

Health care avoidance in the COVID-19 pandemic has been widely reported. Yet few studies have investigated the dynamics of hospital avoidance behavior during pandemic waves and inferred its impact on excess non–COVID-19 deaths.

**Objective:**

This study aimed to measure the impact of hospital avoidance on excess non–COVID-19 deaths in public hospitals in Hong Kong.

**Methods:**

This was a retrospective cohort study involving 11,966,786 patients examined between January 1, 2016, and December 31, 2021, in Hong Kong. All data were linked to service, treatment, and outcomes. To estimate excess mortality, the 2-stage least squares method was used with daily tallies of emergency department (ED) visits and 28-day mortality. Records for older people were categorized by long-term care (LTC) home status, and comorbidities were used to explain the demographic and clinical attributes of excess 28-day mortality. The primary outcome was actual excess death in 2020 and 2021. The 2-stage least squares method was used to estimate the daily excess 28-day mortality by daily reduced visits.

**Results:**

Compared with the prepandemic (2016-2019) average, there was a reduction in total ED visits in 2020 of 25.4% (548,116/2,142,609). During the same period, the 28-day mortality of non–COVID-19 ED deaths increased by 7.82% (2689/34,370) compared with 2016-2019. The actual excess deaths in 2020 and 2021 were 3143 and 4013, respectively. The estimated total excess non–COVID-19 28-day deaths among older people in 2020 to 2021 were 1958 (95% CI 1100-2820; no time lag). Deaths on arrival (DOAs) or deaths before arrival (DBAs) increased by 33.6% (1457/4336) in 2020, while non–DOA/DBAs increased only by a moderate 4.97% (1202/24,204). In both types of deaths, the increases were higher during wave periods than in nonwave periods. Moreover, non-LTC patients saw a greater reduction in ED visits than LTC patients across all waves, by more than 10% (non-LTC: 93,896/363,879, 25.8%; LTC: 7,956/67,090, 11.9%). Most of the comorbidity subsets demonstrated an annualized reduction in visits in 2020. Renal diseases and severe liver diseases saw notable increases in deaths.

**Conclusions:**

We demonstrated a statistical method to estimate hospital avoidance behavior during a pandemic and quantified the consequent excess 28-day mortality with a focus on older people, who had high frequencies of ED visits and deaths. This study serves as an informed alert and possible investigational guideline for health care professionals for hospital avoidance behavior and its consequences.

## Introduction

The COVID-19 pandemic has posed challenges to population health beyond infection. On one hand, viral infection is a major health threat to vulnerable patients; on the other hand, among all patients, hospital non–COVID-19 mortality increased significantly. A retrospective, multicenter study in the United States reported that 30-day risk-adjusted non–COVID-19 mortality has increased by more than 20% since the pandemic [1].

Emergency departments (EDs) play an important role in the management of patients infected with COVID-19 and those with other medical emergencies. It is the first point of contact for many COVID-19 patients. EDs play an important role in diagnosis, treatment, and infection control (by isolation or hospitalization, if necessary). However, EDs manage general patients at the same time, meaning that they have to reserve capacity to manage patients with other medical emergencies.

A significant reduction in ED visits has been observed worldwide since the COVID-19 pandemic [1-4]. This reduction may be attributed to either active efforts to avoid contracting the virus in health care facilities or to passive avoidance caused by mandatory lockdown measures [3-5]. Most quantitative studies have reported excess mortality associated with reduced ED visits, although to different degrees [2], and only a few inferred the causative effect [6]. Disease-specific analysis with simpler pandemic dynamics of both excess and reduced mortality have also been reported [7,8], yet the relationships across gender, age groups, and comorbidities remain a significant knowledge gap.

Delayed medical visits and subsequent delayed diagnosis and extended symptomatic periods are most often associated with a time-dependent progression [9-11]. A meta-analysis of delayed cancer care during the pandemic involving 62 studies identified 38 different categories of delays and disruptions with impacts on treatment, diagnosis, or general health services. However, the most frequent determinants for disruptions were provider- or system-related and were caused by reductions in service availability [12-15] rather than patient-related hospital avoidance.

In our previous work, we identified a significant reduction in ED visits in the first 8 months of the COVID-19 pandemic that was associated with an increase in deaths certified in the ED and 28-day mortality in Hong Kong [16,17], despite the service in EDs never being disrupted or reduced. However, the causal effect of this observation has not been established. In this territory-wide retrospective cohort study, we measured the impact of hospital avoidance among ED patients on excess mortality using ED patient data from 2016 to 2021, a period during which Hong Kong experienced a unique COVID-19 pandemic with 4 distinct waves.

## Methods

### Participants and Source of Data

We performed a territory-wide retrospective cohort study using data from the Clinical Data Analysis and Reporting System (CDARS) [18], an administrative clinical database managed by the Hospital Authority of the Hong Kong Special Administrative Region, China. CDARS includes patients’ demographics, death data, diagnoses, procedures, drug prescriptions, dispensing history, and laboratory results from all public hospitals and clinics in Hong Kong. It contains inpatient and outpatient data from over 90% of the 7.47 million people served by the Hong Kong Hospital Authority [19]. The accuracy of diagnostic codes for case identification in CDARS has been validated in previous reports [20-22] with good reliability [23,24].

Full-scale emergency medicine services providing 24-hour, emergency, physician-led care are only available in 18 public hospitals under the hospital authority. In the nonpandemic years of 2016-2019, the total number of visits at these 18 EDs was 8.6 million, equivalent to an average of 287 visits per 1000 population yearly [25].

All patients attending the EDs of the 18 public hospitals in Hong Kong in the prepandemic period of January 1, 2016, to December 31, 2019, and the pandemic period of January 1, 2020, to December 31, 2021, were included. Data extraction took place on February 1, 2022, so all patients were followed up for at least 28 days.

To avoid confounding related to visits by COVID-19–positive patients, all COVID-19–positive visits and inpatient episodes were excluded from analysis. Daily COVID-19 case numbers and confirmed COVID-19 death numbers were obtained from an actively maintained online repository. Data included sex, age, long-term care home (LTC) resident status, ED visit service data, International Classification of Diseases, Ninth Revision, Clinical Modification (ICD-9-CM) codes, and treatments. Among all records, there were 3716 ED visits recorded with positive polymerase chain reaction (PCR) testing results for COVID-19, and these records were excluded from the analysis.

### Ethical Considerations

All patient record entries are anonymized. The institutional review board of the University of Hong Kong/Hospital Authority West Cluster approved the study (UW 20-112) and granted a waiver of participant consent.

### Visit Reduction and 28-Day Mortality

Deaths within 28 days of the last ED visit are defined as 28-day mortality. This was imputed by death registration minus visit date, which must be less than or equal to 28 days. Daily 28-day mortality counts were tallied by the deceased patients’ last visit dates. As visit reduction was hypothesized to take place prior to subsequent excess mortality, counting deaths by visit dates (instead of simply deaths by dates) is preferable. The percentage change in ED visits was analyzed for fair comparison across age and sex strata in each COVID-19 wave and after-wave period.

### Excess 28-Day Mortality Estimation by Causal Inference

In this study, we used the 2-stage least squares (2SLS) method [26] to estimate the daily excess 28-day mortality from daily reduced visits. The 2SLS is a common tool for causal inference. Others have characterized the extent of excess deaths by regression [27], difference-in-difference [28], and propensity matching [29]. Yet these methods cannot infer causation between attendance and death in a population. The 2SLS model is visualized as follows (Multimedia Appendix 1, Figure S1 contains further details):



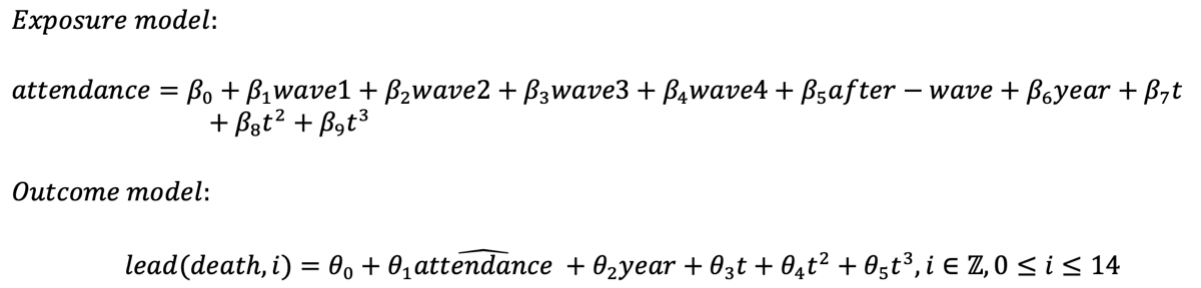



Five binary instrumental variables (IV) or Z were introduced to model the effect of COVID-19 waves on visit reduction: after-wave and wave 1 to wave 4. After-wave IV is 1 for dates after each wave and excludes all COVID-19 waves; otherwise it is 0. IV for waves 1 to 4 are 1 for dates within a COVID-19 wave; otherwise they are 0. Waves 1, 2, 3, and 4 were designated with reference to government actions (Multimedia Appendix 1, Table S1), which were usually implemented at the onset and recession of COVID-19 waves; hence, these act as pivotal timestamps of pandemic severity.

On validity of the wave periods as IVs, calendar time has been reported to be a valid IV [6,7] when dramatic changes in practice occur in a relatively short period of time. As calendar time has been used in COVID-19 literature as an IV [8-10], we believe that government announcements, policy changes, and surges in COVID-19 case numbers from nonwave to wave periods in Hong Kong can also be regarded as drastic changes and are not seasonal (ie, have no L/U-Z association), making the wave periods a valid IV. We also believe that by excluding COVID-19–positive patients from the study population, the association between COVID-19 and 28-day death numbers (ie, Z-Y) has been minimized.

Age and sex strata with mean daily 28-day mortality from 2016 to 2021 less than or equal to 1 were excluded from the causal inference analysis due to model instability. The strata were the age groups 0-17, 18-34, and 35-44 years and both sexes (male and female).

We used the *ivreg* package [11,30] of R (R Foundation for Statistical Computing) to implement 2SLS. First, the exposure (daily number of ED visits) was regressed on the 5 binary IVs: β_1_ to β_5_. Then, we regressed ead (death, i), that is, ED 28-day mortality, with *i* lagged days on the predicted exposure, (ie, attendance), which yielded the local average treatment effect (LATE) among the compliers of each age and gender stratum. In both stages, we added 4 terms for seasonality adjustment: year (2016 was 0), *t* (number of days since the first day of a year), squared *t*, and cubic *t*.

Time lag *i* was also introduced to investigate the lagging effect of visit reduction on future 28-day deaths. For example, the excess death coefficient, θ_1_, can be interpreted as follows: if θ_1_ is –0.02 for a population at a time lag of 5 days, there is a reduction of 100 ED visits on a certain day that caused 2 excess deaths 5 days away. Multimedia Appendix 1, Figures S2A and 2B show the dynamics of visits and deaths estimated by 2SLS models in tandem with COVID-19 waves.

To establish the validity of using COVID-19 waves as instrumental variables, a partial *F* statistic test was conducted between instruments and exposure [31] (visit with no time lag).

### Individual-Level Longitudinal Analysis of the Incidence Rate of Deaths on Arrival or Deaths Before Arrival Among Older People

Deaths on arrival (DOAs) or deaths before arrival (DBAs) among 2 groups of people (COVID-19 and pre–COVID-19) aged 65 years or older were identified by patient IDs and filtered by those who visited at least twice in their respective control periods. The COVID-19 DOA/DBA group of older people visited EDs from between July 1, 2020, and July 1, 2021. Changes in their individual incidence rates (daily ED visits) between January 1, 2019, and January 24, 2020, (the control period) and between January 25, 2020 (the pandemic start), and June 30, 2020 (the treatment period), were calculated. The pre–COVID-19 DOA/DBA group of older people visited EDs between July 1, 2018, and July 1, 2019. Changes in their individual incidence rates between January 1, 2017, and June 30, 2017 (the control period), and January 1, 2018, and June 30, 2018 (the treatment period), were calculated.

### DOA/DBAs and Comorbidities Among Older People

To further investigate clinical attributes of the excess 28-day mortality among older patients, we divided patient mortality in EDs into DOA/DBAs and non-DOA/DBAs.

Among DOA/DBAs, most had no ICD-9-CM diagnosis code or were simply diagnosed as, for example, cardiac arrest, so their comorbidities cannot be inferred meaningfully. For non-DOA/DBAs, their diagnostic codes were mapped to Deyo comorbidities using the R package *icd* [32]. Episode ED admissions and 28-day mortality were counted for each selected comorbidity.

## Results

We examined a total of 11,966,786 ED visit records from January 1, 2016, to December 31, 2021, among which there were 212,288 (1.8%) patient deaths within 28 days of their last visit to the ED. We found that 28-day ED mortality was 43,335 in 2020 and 44,205 in 2021, compared to the 2016-2019 average of 40,192 (Table 1).

**Table 1:** Yearly changes in absolute numbers of and ratios between total 28-day emergency department mortalities and registered all-cause deaths in Hong Kong government censuses; the values include the 2016-2019 average and values for 2020 and 2021.

Time period	28-day ED^a^ mortalities, n	Increase in 28-day ED mortalities vs 2016-2019 average, n	Registered all-cause deaths, n	Increase in registered all-cause deaths vs 2016-2019 average, n	28-day ED mortalities/registered all-cause deaths, %	Increase in 28-day ED mortalities/registered all-cause death vs 2016-2019 average, %
2016-2019 average	34,370	—^b^	47,523	—	72.3	—
2020	37,059	2689	50,666	3143	73.1	0.8
2021	37,662	3292	51,536	4134	73.1	0.8

^a^ED: emergency department.

^b^Not applicable.

### Visit Reductions and 28-Day Mortality

**Figure 1:**
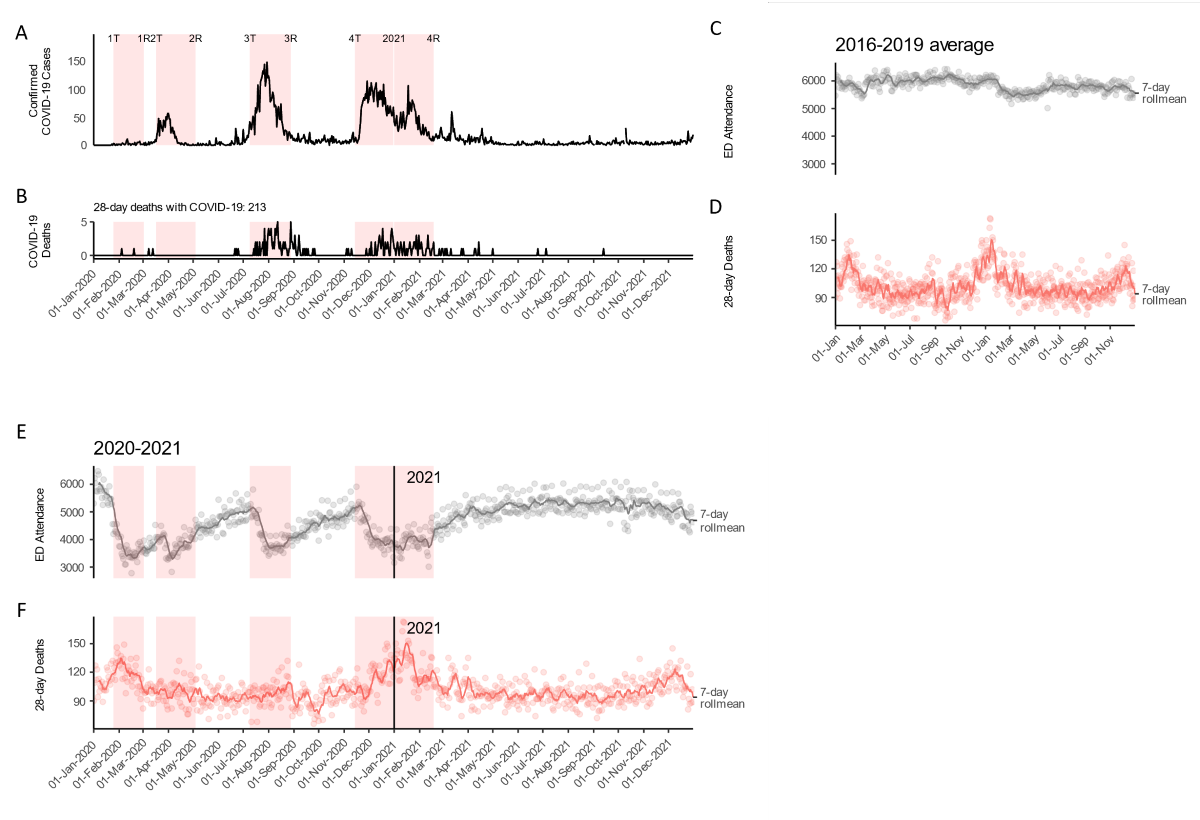
Emergency department (ED) visits and 28-day mortality at all Hong Kong public hospital EDs during 2016 to 2019 and during the COVID-19 pandemic period (2020-2021). (A) Daily confirmed COVID-19 cases and (B) official COVID-19 mortality, with waves shaded pink. Waves are defined by government-mandated tightening or relaxation of antipandemic measures. (C) Average daily ED visits and (D) 28-day deaths from 2016 to 2019. (E) Daily ED visits and (F) 28-day deaths in 2020 and 2021.

Compared with the average in 2016-2019, there was a reduction in total ED visits in 2020 of 25.4% (548,116/2,142,609). Total ED visits were 1,594,493 in 2020 and 1,812,703 in 2021, compared to the 2016-2019 average of 2,142,609 (Multimedia Appendix 1, Table S2). During the same period, the 28-day mortality of non–COVID-19 ED deaths increased by 7.82% (3143/40,192) compared with 2016-2019 (Table 1). The COVID-19 pandemic progressed in Hong Kong through 4 distinct waves, with the fourth wave lasting until February 18, 2021, resulting in 149 COVID-19 deaths in 2020 and 64 in 2021. ED visits in 2016-2019 was roughly stable throughout the year, while in 2020 and 2021 they fluctuated and mostly showed noticeable decreases at the start of each wave and subsequent resurgences (Figures 1A and 1B). These decreases in ED visits in 2020 and 2021 generally correspond to an increase in excess ED deaths, particularly during the first and fourth waves (Figures 1E and 1F).

The number of ED visits and the 28-day mortality of ED patients in 2016-2019 and in 2020 and 2021 are shown in Figures 1C to 1F. Compared with the 2016-2019 average value in Figure 1C, the number of visits in 2020 and 2021 demonstrated an observable reduction, to between 3000 and 4000 daily, during each wave of COVID-19, followed by a gradual rebound to a higher visit number until the next wave. With relatively improved infection control in 2021, the number of visits reached close to prepandemic levels in late 2021 (Figure 1E). The 28-day mortality in 2020 roughly followed the pattern of the 2016-2019 average until the fourth wave, during which the mortality reached its peak throughout the study period (Figure 1D and 1F). The median ED waiting time from entry to reaching a cubicle in 2020 displayed similar dynamics as the ED visit curve (Multimedia Appendix 1, Figure S3).

### Excess 28-day Mortality Estimation by Causal Inference

**Figure 2:**
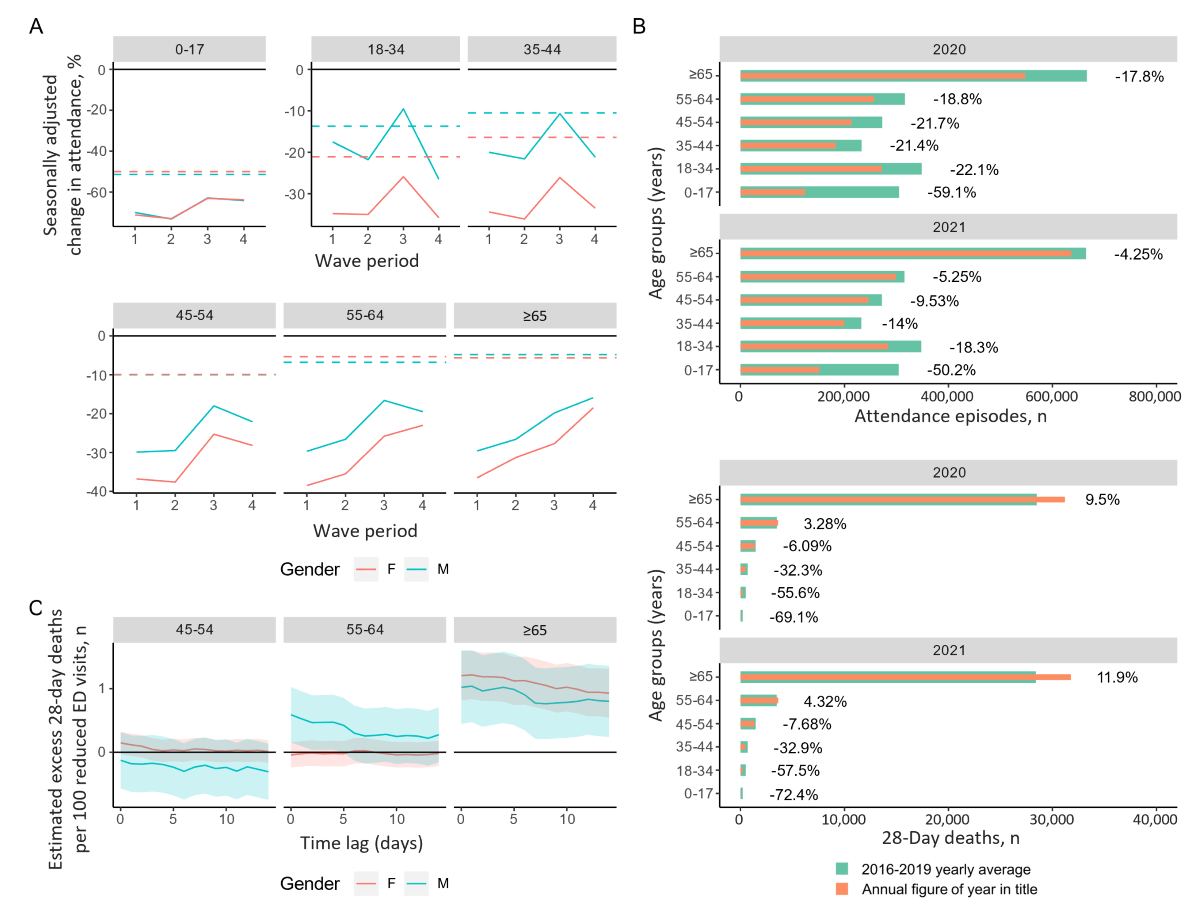
Seasonally adjusted changes in Hong Kong public hospital emergency department visits and 28-day mortality, segmented by age and sex, from 2016 to 2019 and in 2020 and 2021, per wave period and yearly. (A) Line plots of seasonally adjusted visit change percentage among age and sex groups across waves, with after-wave periods shown by dashed lines (see Methods). The seasonally adjusted visit change percentage is calculated as the same-period change percentage against the 2016 to 2019 average. (B) Emergency department visits and excess 28-day deaths across age strata with percentage changes between 2020 and the 2016 to 2019 average, as well as 2021 vs the 2016 to 2019 average. Numerical values are percentage changes from the 2016 to 2019 average to the annual sum of each year (2020 and 2021). (C) Line plots of excess 28-day mortality per 100 reduced emergency department visits among 45-54, 55-64, and ≥65 year age groups and sex groups (95% CIs are shown by the shaded areas) across 0-to-14-day delays. This was estimated by instrumental variable analysis, with wave and after-wave periods as instrument variables, emergency department attendance as exposure, and emergency department 28-day deaths as outcome.

Next, the reduced ED visits and 28-day excess mortality across age and sex groups were characterized. Visits in each wave period were adjusted to same-period 2016-2019 data, while after-wave period data across 2020 and 2021 were summed and adjusted. In all adult groups, female patients saw a larger reduction in annualized visits than male patients of the same age in every wave (Figure 2A). However, it is also notable that in the 55-to-64 and ≥65-year age groups, the patients were less affected by further waves with time. ED visits and excess 28-day deaths across age strata with percentage changes from the 2016-2019 average and 2020 and 2021 are shown in Figure 2B. The reduction of ED visits was attenuated in 2021 when compared with 2020, but it was not restored to the 2016-2019 average.

Although the ≥65-year age group had the highest absolute numbers of ED visits in the study period (3,849,923 episodes; Multimedia Appendix 1, Table S1), the 0-to-17-year age group had by far the largest reduction in ED visits in 2020 and 2021 from the 2016-2019 average (range –49.4% to –59.3%, n=–65,232/132,330; –102,411/172,781; Figure 2B and Multimedia Appendix 1, Table S3).

Apart from being the largest contributor to the total number of ED visits, the older group also saw the largest excess 28-day mortality in 2020 and 2021 (Figure 2C). Older male patients had a similar excess 28-day mortality rate (with a 95% CI greater than 0) to female patients, with an immediate (no time lag) excess 28-day death rate of 0.953 (95% CI 0.382-1.52) per 100 reduced ED visits among male patients and 1.15 (95% CI 0.763-1.54) among female patients (Multimedia Appendix 1, Table S4). All partial *F* statistics had a *P* value <.05.

Multimedia Appendix 1, Table S5 provides estimates of older male and female total excess deaths by visit reduction in 2020 and 2021. The estimated total excess non–COVID-19 28-day deaths due to reduced ED visits among older people throughout 2020 and 2021 was 1958 (95% CI 1100-2820; no time lag). The actual excess deaths in 2020 and 2021 were 3143 and 4013, respectively, with the 2016-2019 average in the census [33,34] as the benchmark.

### Individual-Level, Longitudinal Analysis of Incidence Rate of DOA/DBAs Among Older People

The ED excess 28-day mortality of older patients can be categorized as DOA/DBAs and non-DOA/DBAs. DOA/DBAs increased by 1457 or 35.1% in 2020, while non-DOA/DBA mortalities increased by only a moderate 1202 or 4.65% (Multimedia Appendix 1, Table S6). Table 2 provides a breakdown of the changes in both DOA/DBAs and non-DOA/DBAs; the increases were higher during wave periods than in nonwave periods. The corresponding results in 2021 generally follow the same trends as the ones in 2020.

**Figure 3:**
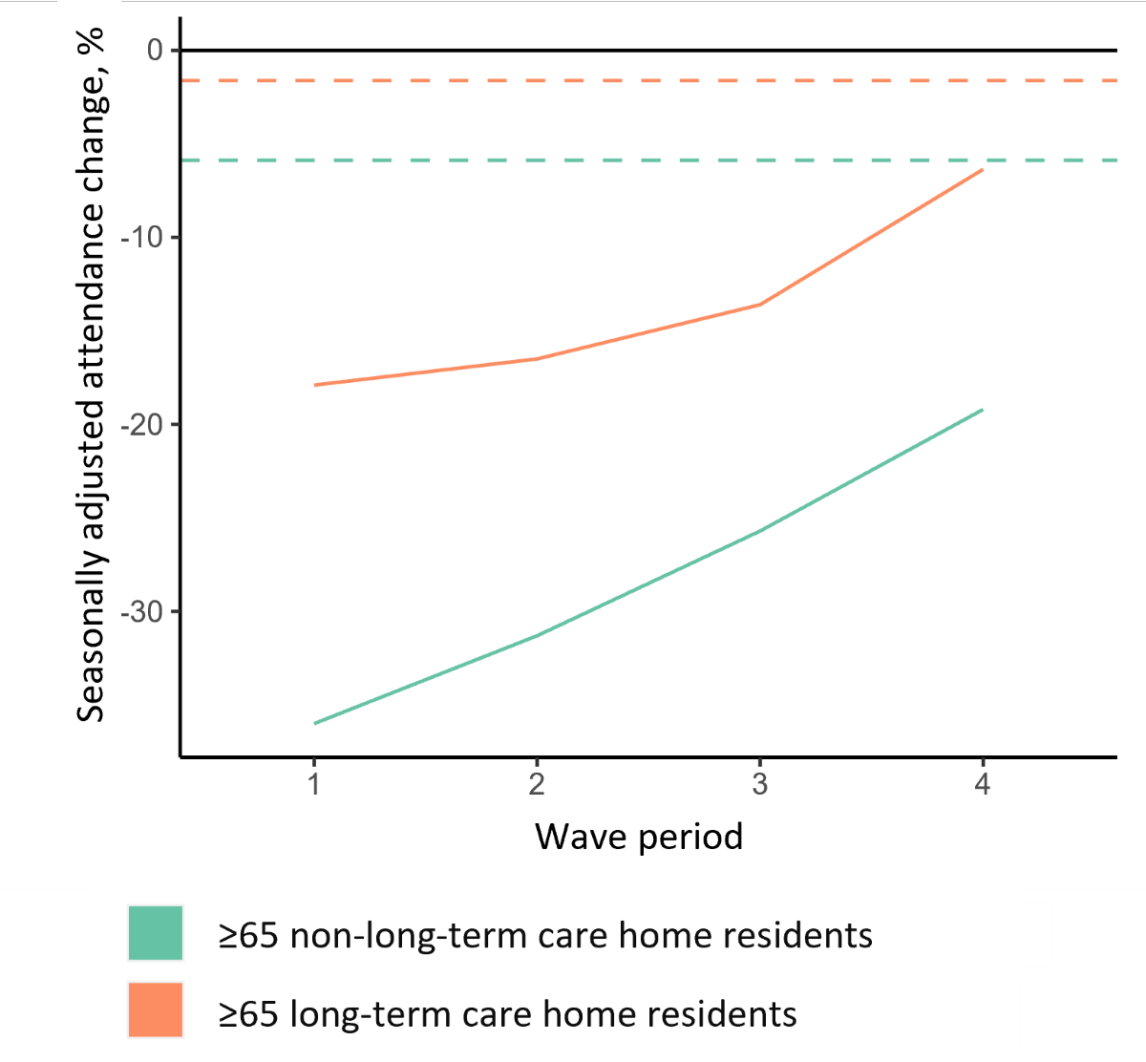
Seasonally adjusted change (vs 2016-2019 average) in public hospital emergency department visits across waves, with the after-wave period in dashed lines.

Results in Table 3 show that there were more DOA/DBAs among older people without ED visits or who reduced their visits in the COVID-19 group when compared to their number of ED visits at least twice in prior months. Moreover, non-LTC patients saw a greater reduction in ED visits than LTC patients across all waves by more than 10% (non-LTC: 93,896/363,879, 25.8%; LTC: 7956/67,090, 11.9%) (Figure 3). We further broke down DOA/DBA and non-DOA/DBA mortalities among older people by their LTC residence status (Table 4). In 2020, DOA/DBAs among non-LTC residents increased by 42.4% (1284/3026), which is a more than 7-fold greater increase compared to that among LTC residents (173/1310, +13.2%). In 2021, the trends were similar, apart from a smaller LTC death increase. Taken together, these results show that non-LTC patients had a much higher rate of DOA/DBA excess ED deaths during the COVID-19 pandemic (when normalized against 2016-2019).

**Table 2:** Excess emergency department 28-day deaths in the ≥65-year age group, broken down by deaths on arrival or deaths before arrival (DOA/DBA) status; wave and nonwave periods in 2020 and 2021, as defined in Multimedia Appendix 1, Table S1; and same-period 2016-2019 average deaths.

Year			2016-2019 average deaths, n		Change, n (%)
**2020**					
	Non-DOA/DBA, nonwave (179 days)	11,486		565 (4.92)	
	Non-DOA/DBA, wave (186 days)	12,717		561 (4.41)	
	DOA/DBA, nonwave	1973		571 (28.9)	
	DOA/DBA, wave	2316		933 (40.3)	
**2021**					
	Non-DOA/DBA nonwave	20,278		1815 (8.95)	
	Non-DOA/DBA wave	3852		345 (8.96)	
	DOA/DBA nonwave	3508		895 (25.5)	
	DOA/DBA wave	765		301 (39.3)	

**Table 3:** Same-period comparison of the incidence rate of deaths on arrival/deaths before arrival (DOA/DBAs) among people aged ≥65 years during COVID and before COVID. All DOA/DBAs were among people with at least 1 emergency department visit during the control period. Increases and reductions in the incidence rate describe the number of DOA/DBAs among people aged ≥65 years who had increased or reduced individual incidence rates in the treatment period compared to the control period. “No visits in treatment period” describes the number of DOA/DBAs among people aged ≥65 years without emergency department visits during the treatment period. *P* values were derived from a 2-sample z test.

	DOA/DBAs among people aged ≥65 years during COVID-19^a^	DOA/DBAs among people aged ≥65 years before COVID-19^b^	*P* value
Increase in incidence rate (n=416)	194	222	.053
No visits in treatment period (n=628)	427	201	<.001
Reduction in incidence rate (n=542)	307	235	<.001

^a^Between July 1, 2020, and July 1, 2021; control period: January 1, 2019, to December 31, 2019; treatment period: January 25, 2020, to June 30, 2020.

^b^Between July 1, 2018, and July 1, 2019; control period: January 1, 2019, to December 31, 2019; treatment period: January 1, 2018, to June 30, 2018.

**Table 4:** Seasonally adjusted change in 28-day emergency department mortality among people aged ≥65 years by long-term care (LTC) and death on arrival/death before arrival (DOA/DBA) status.

Year		Seasonally adjusted change, n (%)
**2020**		
	LTC, non-DOA/DBA (n=8760)	524 (5.98)
	LTC, DOA/DBA (n=1310)	173 (13.2)
	Non-LTC, non-DOA/DBA (n=15,444)	678 (4.39)
	Non-LTC, DOA/DBA (n=3026)	1284 (42.4)
**2021**		
	LTC, non-DOA/DBA (n=8760)	806 (9.20)
	LTC, DOA/DBA (n=1310)	94 (7.18)
	Non-LTC, non-DOA/DBA (n=15,444)	1366 (8.84)
	Non-LTC, DOA/DBA (n=3026)	1055 (34.9)

### DOA/DBAs and Comorbidities Among Older People

**Figure 4:**
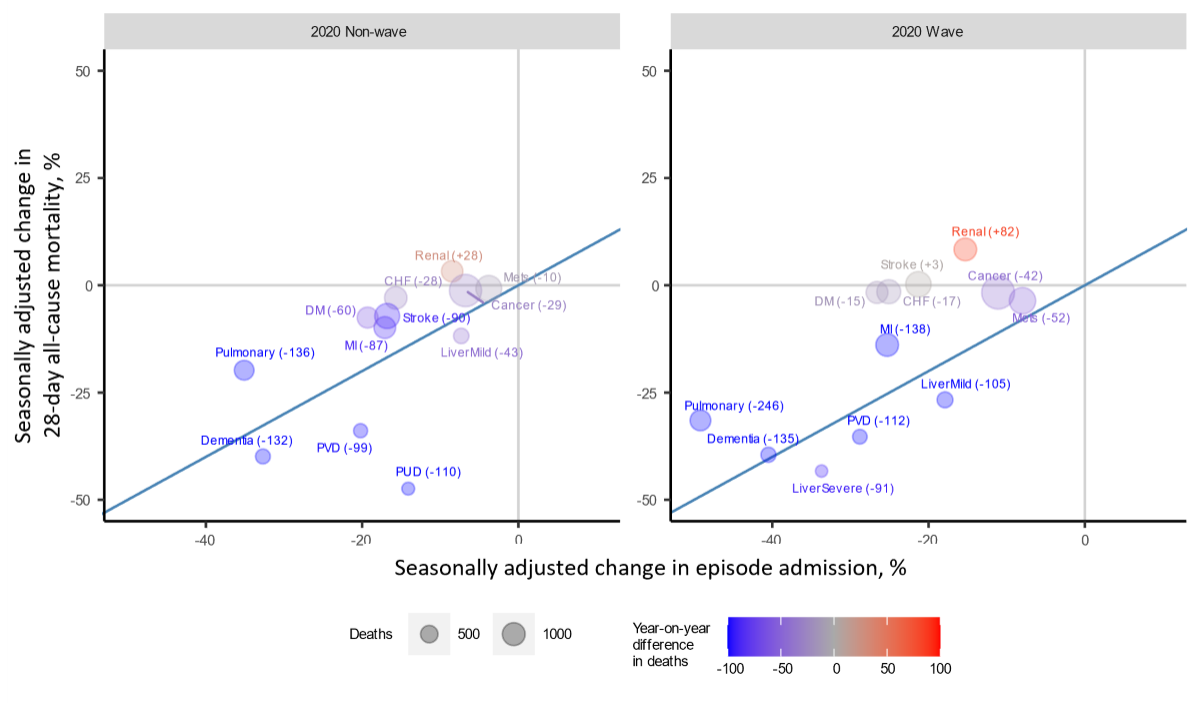
Seasonally adjusted change in episode admission among people aged ≥65 years by comorbidity and 28-day all-cause mortality, as well as absolute yearly change in 28-day mortality, vs the 2016-2019 average in 2020 wave and nonwave periods. The blue line is the 1:1 visit and death change line, on which high mortality-rate situations like cardiac arrest and sudden death normally fall. Deaths on arrival/deaths before arrival were excluded. Comorbidities were defined by the Charlson comorbidities. CHF: congestive heart failure; DM: diabetes mellitus; MI: myocardial infarction; PUD: peptic ulcer disease; PVD: peripheral vascular disease.

Most of the comorbidity subsets demonstrated an annualized reduction in visits in 2020, as shown in Figure 4. Patients diagnosed with renal diseases and severe liver diseases saw a notable increase in deaths.

## Discussion

### Principal Findings

This study used 2SLS modeling to quantify hospital avoidance behavior and resulting excess mortality in the COVID-19 pandemic across sex, age groups, and comorbidities. Through causal inference, we discovered a higher prevalence of hospital avoidance behavior in female patients, children, and adolescents during the study period. Although surges of COVID-19 patients may overwhelm health systems and increase excess 28-day deaths [35], the waiting time in EDs in 2020 did not vastly differ from the 2016-2019 average in Hong Kong (Multimedia Appendix 1, Figure S3) [36]. Thus, the capacity of ED service was not severely impacted in 2016-2021 and we can exclude the effect of ED capacity on excess death.

The association between COVID-19 waves and hospital avoidance was observed among all age and sex groups. However, the extent of reduction varied in each age group. Lange et al [37] reported a similarly greater age-related reduction in ED use under the National Syndromic Surveillance Program for patients with selected comorbidities. On the other hand, Hung et al [38] did not discover any statistically significant differences in hospital avoidance among different age groups in their survey.

Stratified by sex, we found that women avoided hospitals more than men. This concurs with the literature on hospital avoidance behavior during the pandemic [36-40].

The largest relative reduction in ED visits in this study was found in children and adolescents (Figure 2A, Multimedia Appendix 1, Table S2), which is consistent with previous reports. Parents or caretakers may weight infection risk more heavily than the risk of delayed illness management. They might resort to visiting clinics rather than the ED or even adopt home care, given that children and adolescents are the healthiest of all age groups.

At a population level, there was a statistically significant excess death rate among older people associated with reduced ED visits. The results in Table 3 show that at the individual level, there was a significant difference in hospital avoidance behavior among older people during the pandemic compared to the prepandemic period. These observations imply that in Hong Kong, excess deaths among older people confirmed by the ED were more often due to the individual’s decision to avoid hospitals and stay home despite being severely ill rather than because of worsened hospital treatment received during the pandemic. Further breakdown of the results (Multimedia Appendix 1, Tables S6 and S7) by LTC status shows that there was a significant decrease in non-LTC ED visits during the pandemic, and there were nearly half the number of non-LTC ED DOA/DBAs among older people (n=280) compared to their LTC counterparts (n=147) (Multimedia Appendix 1, Tables S8 and S9). It is therefore evident that older people who were prone to sudden death were less likely to visit EDs during the pandemic. LTC residents receive more attention in general, and their ED visits were thus less affected. The increase in DOA/DBAs was also more drastic in the non-LTC population than the LTC population, suggesting that health monitoring and advice to obtain medical consultation benefited their health during the pandemic.

The examination of deaths among older people by LTC status offers insight into how daily care or the lack thereof affects health outcomes. While COVID-19 waves intensified hospital avoidance behavior among older people both in and out of LTC, older people out of LTC were much more reluctant to go to hospitals in all periods (Multimedia Appendix 1, Table S7). The increase in DOAs among non–LTC residents was at least 7-fold higher than that among LTC residents (Multimedia Appendix 1, Table S6), which is an alarming difference. It can be inferred that daily care has an important role in mitigating disease progress and preventing excess deaths. Older people outside LTC, living either alone or with their families, might have received less attention or professional advice during the pandemic, leading to less-prompt ED visits. Conversely, LTC residents are monitored by staff, which includes nurses. LTC residents were more likely to get on-time ED care when in need and therefore were more likely to be encouraged by medical professionals or social workers to seek ED care.

We studied the clinical characteristics of the older patients, as well as the relationships of these characteristics with ED visit reduction and changes in death at different time points of entering the ED. Patients with all filtered Deyo comorbidities saw visit reduction, showing that hospital avoidance behavior is universal, though its extent differs, in line with reports from the United States [40]. With respect to patients with acute conditions, visit reduction among patients with ischemic heart diseases was close to those with stroke, ranging from 15% to 25% in both sexes and in the 2 periods. The specific comorbidities that saw a notable rise in deaths overlaps with the leading causes of death in Hong Kong [41]. One seemingly contradictory observation is that patients with ischemic heart diseases did not show excess 28-day deaths, although researchers have reported otherwise. We postulate that patients who delayed ED visits developed cardiac arrest before arrival, which would not have been captured by the diagnosis or death codes.

The simplicity of our model is the result of a balancing act between the accurate depiction of hospital avoidance behavior and the minimization of bias. Various strategies to accurately model hospital avoidance behavior have been attempted yet rejected on the ground of interpretability and possible statistical bias. Though the data are comprehensive for the patients’ ED diagnoses and hence their clinical severity, we still lack a core measure of delayed treatment (ie, the delay time duration from symptom onset to ED), which is more common in studies related to stroke. In this regard, we can only infer the consequences of delayed treatment. Other limitations include data coverage (the absence of private hospital data), statistical method (dynamic models), and clinical data (survivors’ outcome or vital data). For IV analysis, various assumptions have to be made that also lead to limitations. One is that IV is associated with exposure, which has been bolstered by *F* statistics showing that the association is significant. Another assumption is that IV does not affect the outcome except through its potential effect on exposure, which can be quite challenging to confirm, as the COVID-19 pandemic impacted many aspects of life. The pandemic might have indirectly caused mortality, such as through its association with increased suicidal ideation, which could have caused us to overestimate the impact of ED attendance reduction on excess deaths. Yet territory-wide death numbers in government reports are higher than the ED deaths reported in our study. The discrepancy, part of which was probably caused by delays in or avoidance of hospital care, cannot be elucidated by our study and may lead to an underestimation of the true impact. IV and the outcome should not share causes; this requirement has been partially satisfied by removing COVID-19–positive patients from the data, such that the remainder of ED visits were less likely to be impacted by COVID-19. The last assumption is monotonicity. It has been discovered that women perceived the COVID-19 pandemic as a greater threat and were more likely to avoid health care than men [15,16]. Younger groups have also been reported to have had a greater tendency to avoid needed care during the pandemic [17,18]. Both of these results coincide with our findings. We hoped to satisfy the monotonicity assumption by dividing the population into age and sex strata, where people in the same strata are expected to have similar tendencies in avoiding hospital care.

Despite the limitations, our study used ED data during the local COVID-19 pandemic with tidal characteristics clearer than those for other countries or regions. It offers a chance to gauge event-driven hospital avoidance behavior and its toll.

### Conclusion

We demonstrated a statistical method to estimate hospital avoidance behavior during a pandemic and quantified the consequent excess 28-day mortality with a focus on older people, who have a high frequency of ED visits and deaths. This study serves as an informed alert and possible investigational guideline for health care professionals on hospital avoidance behavior and its consequences.

## Appendix

Multimedia Appendix 1 Supplementary Tables S1-S9 and Figures S1-S4.

## Abbreviations

2SLS: 2-stage least squares
CDARS: Clinical Data Analysis and Reporting System
CHF: congestive heart failure
DM: diabetes mellitus
DOA/DBA: death-on-arrival/death-before-arrival
ED: emergency department
ICD-9-CM: International Classification of Diseases, Ninth Revision, Clinical Modification
IV: instrumental variable
LATE: local average treatment effect
LTC: long-term care
MI: myocardial infarction
PCR: polymerase chain reaction
PUD: peptic ulcer disease
PVD: peripheral vascular disease

## Data Availability

A deidentified subject-level data set will be made available on a case-by-case basis on reasonable request to the corresponding author for research purposes 6 months after publication.
