# Supplementary material for: The Effect of the COVID-19 Pandemic on Non–COVID-19 Deaths: Population-Wide Retrospective Cohort Study
Source: JMIR Public Health Surveill. 2024 Feb 13;10:e41792. doi: 10.2196/41792 (PMC10866203; doi:10.2196/41792)
Supplement: Multimedia Appendix 1 [file publichealth_v10i1e41792_app1.docx]

**Supplementary Material**

**Figure S1** Directed acyclic graph (DAG) of instrumental variable/two-stage least squares analysis.

U

Unmeasured confounder


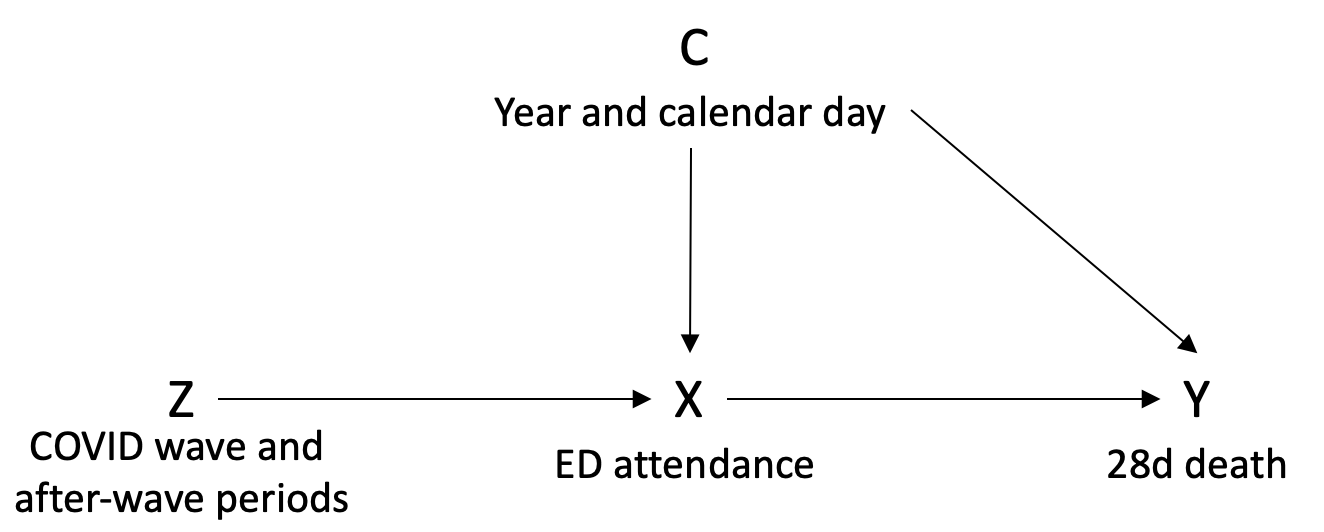


**Equations**

$$Same-period 2016-2019 average attendance or 28d mortality$$

$$=\sum_{i=first day of a period in 2016-2019}^{last day of a period in 2016-2019} {Daily Average}_{i}$$

$$=\sum_{i=first calendar day}^{last calendar day of a period} mean({Daily Average}_{i}, no. of years of i^{th} calendar day)$$

$$Seasonally-Adjusted Attendance Change \% within a period$$

$$=(\frac{Cumulative attendance in 2020/2021 within a period}{Same-period 2016-2019 average attendance}-1)\times100\%$$

$$=(\frac{\sum_{i=first day of a period in 2020 or 2021}^{last day of a period in 2020 or 2021} {Daily Attendance}_{i}}{Same-period 2016-2019 attendance}-1)\times100\%$$

$$Estimated 28d Mortality Change by 1 reduced ED visit$$

$$= -\hat{\theta}_{1}\sim\hat{\frac{\Delta death}{-\Delta\hat{attendance}}}$$

$$Estimated Excess 28d Mortality by 100 reduced ED visits$$

$$=Excess Death Death Rate$$

$$= -\hat{\theta}_{1}\times100$$

For comorbidity plots during wave or non-wave periods:

Wave = waves 1-4; Non-wave = not waves 1-4 including pre-pandemic period

$$Episode Admission Yearly or Seasonally-Adjusted Change \%$$

$$=\left( \frac{\sum_{2020 or 2021} Daily Attendance}{Same-period 2016-2019 attendance}-1 \right)\times100\%$$

$$28d All-Cause Mortality Yearly or Seasonally-Adjusted Change \%$$

$$=\left( \frac{\sum_{2020 or 2021} Daily 28d Mortality}{Same-period 2016-2019 28d mortality}-1 \right)\times100\%$$

$$Absolute Yearly or Seasonally-Adjusted Change of 28d Mortality$$

$$=\sum_{2020 or 2021} Daily 28d Mortality- \sum_{same-period 2016-2019} Daily Average 28d Mortality$$

**Figure S2A, B** Plots of ≥65 male and female actual(dots) and estimated(lines) daily ED visit counts and 28d ED death counts, in 2016-2019 average, 2020 and 2021.


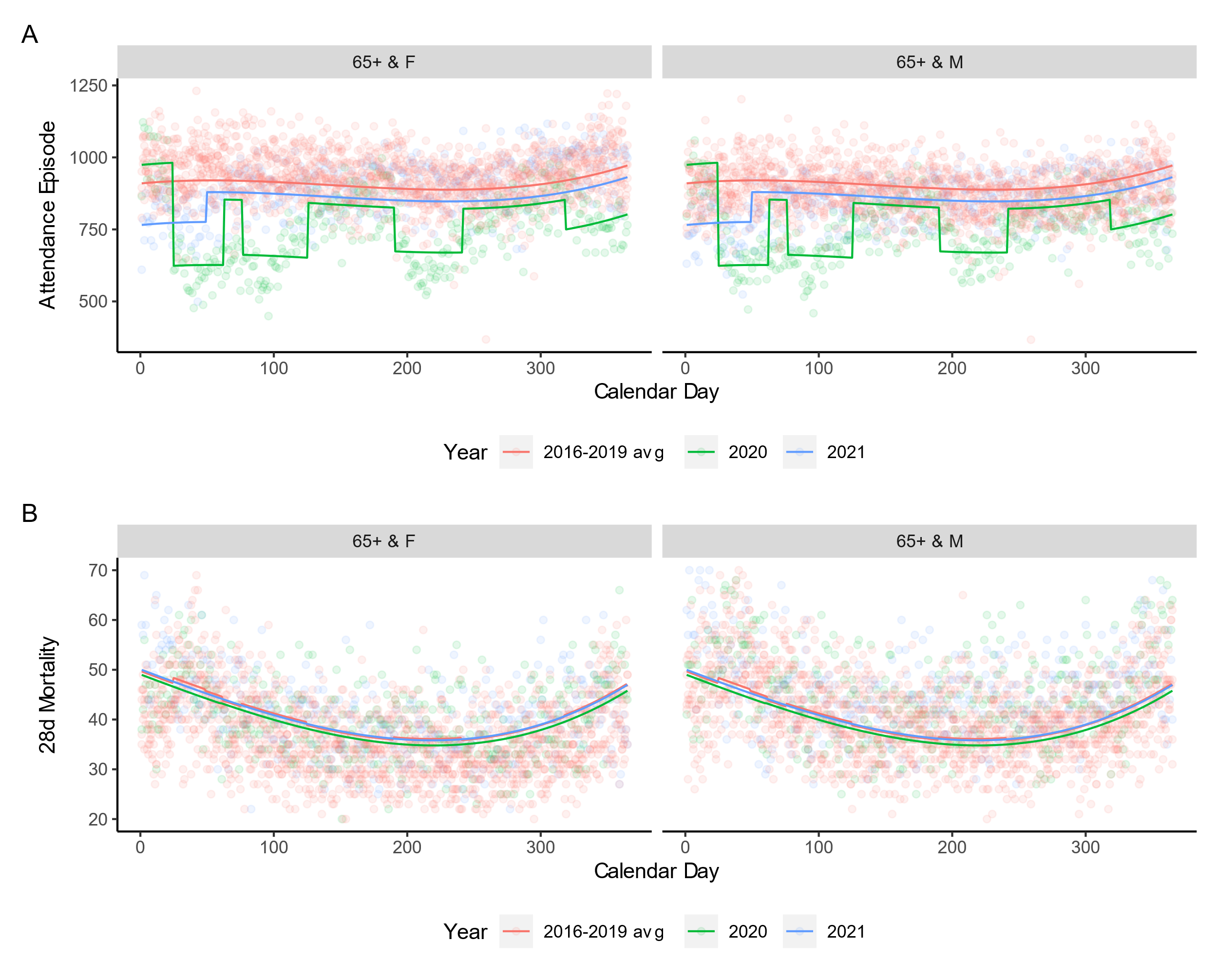


**Figure S3** Median Waiting Time (min) from entry to cubicle between 2016-2019 average (left), and 2020 and 2021 (right), in triage 1-3 levels and triage 4-5 levels. COVID waves 1-4.


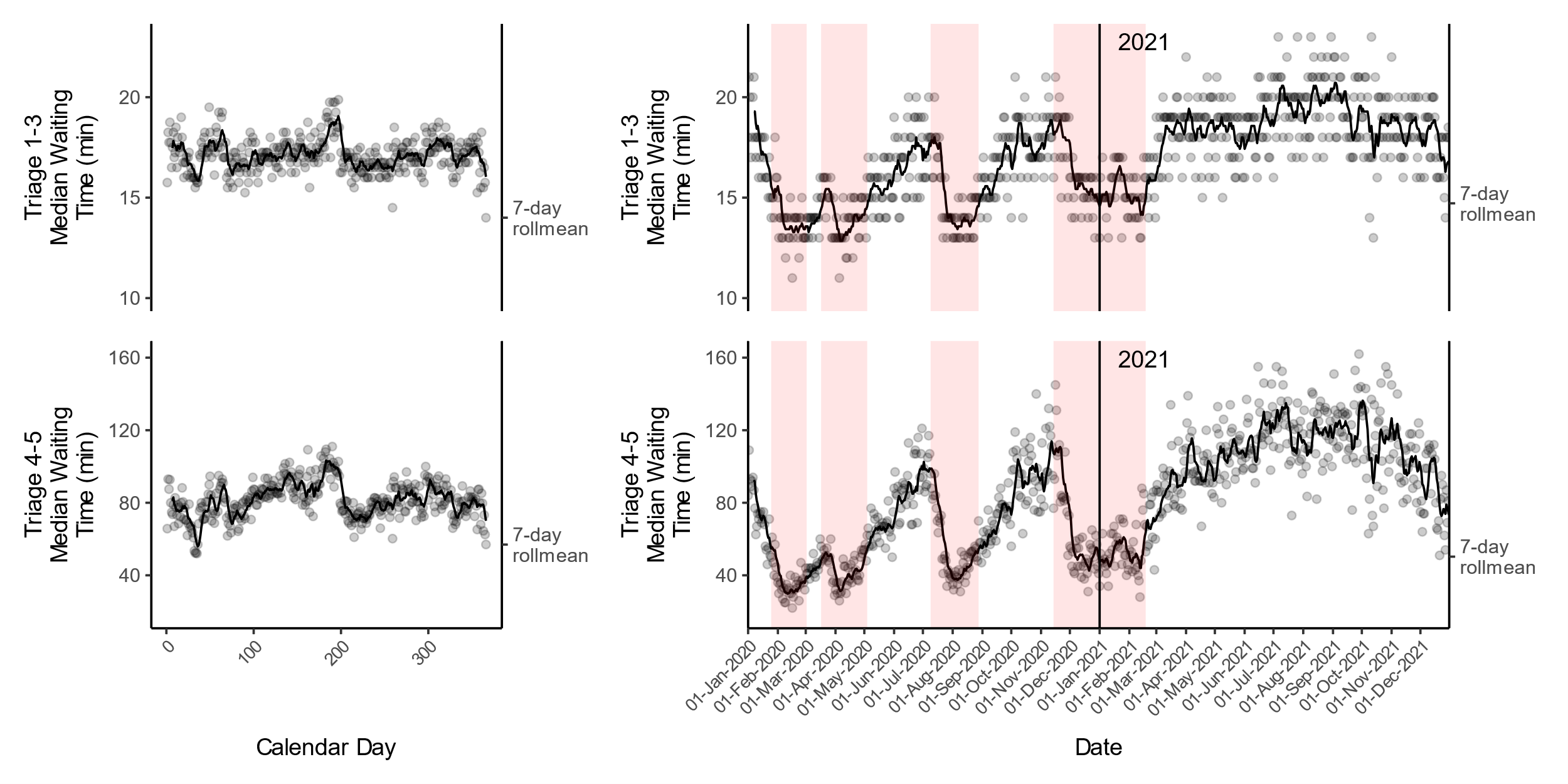


**Table S1** HK Government Action Timeline.

| Date | Wave | Government Actions |
| --- | --- | --- |
| 31^st^ Dec 2019 | / | HA started Enhanced Laboratory Surveillance Programme (ELSP) to detect suspected/confirmed COVID cases^1^ |
| 22^nd^ Jan 2020 | / | First COVID case confirmed in HK^2^ |
| 25^th^ Jan 2020 | 1 (onset) | HK Hospital Authority raised Emergency Response Level to Urgent in response to COVID |
| 19^th^ Feb 2020 | / | ELSP expanded to cover General out-patient Clinics and ED^1^ |
| 2^nd^ March 2020 | 1 (recession) | Resumption of government services with social distancing protocol in place^3^ |
| 17^th^ March 2020 | 2 (onset) | HK Government raised travel alert to red worldwide except Mainland, Macau and Taiwan^4^ |
| 4^th^ May 2020 | 2 (recession) | Resumption of government services, Work-from-home order lifted for government officials^5^ |
| 9^th^ July 2020 | 3 (onset) | Tightening of gathering restrictions^6^ |
| 28^th^ August 2020 | 3 (recession) | Relaxation of gathering and business restrictions, and mask mandate^7^ |
| 14^th^ November 2020 | 4 (onset) | Tightening restrictions in restaurant sitting and opening hours, and social distancing^8^ |
| 18^th^ Feb 2021 | 4 (recession) | Government may ease social distancing^9^ |

**Table S2** Summary of ED visit of various age and gender groups between 2016-2019 average, 2020 and 2021 respectively.

| Year | Age and Gender | 2016-2019 average | Change | Change (%) |
| --- | --- | --- | --- | --- |
| 2020 | 0-17 & F | 132330 | -78284 | -59.2 |
| 2020 | 0-17 & M | 172781 | -102411 | -59.3 |
| 2020 | 18-34 & F | 186170 | -51236 | -27.5 |
| 2020 | 18-34 & M | 162503 | -26384 | -16.2 |
| 2020 | 35-44 & F | 131692 | -35225 | -26.7 |
| 2020 | 35-44 & M | 101482 | -15410 | -15.2 |
| 2020 | 45-54 & F | 156311 | -37612 | -24.1 |
| 2020 | 45-54 & M | 116687 | -22186 | -19 |
| 2020 | 55-64 & F | 159219 | -34446 | -21.6 |
| 2020 | 55-64 & M | 157169 | -25610 | -16.3 |
| 2020 | ≥65 & F | 342167 | -68318 | -20 |
| 2020 | ≥65 & M | 324098 | -50994 | -15.7 |
| 2021 | 0-17 & F | 132330 | -65232 | -49.3 |
| 2021 | 0-17 & M | 172781 | -87625 | -50.7 |
| 2021 | 18-34 & F | 186170 | -39946 | -21.5 |
| 2021 | 18-34 & M | 162503 | -23975 | -14.8 |
| 2021 | 35-44 & F | 131692 | -21468 | -16.3 |
| 2021 | 35-44 & M | 101482 | -11365 | -11.2 |
| 2021 | 45-54 & F | 156311 | -14497 | -9.27 |
| 2021 | 45-54 & M | 116687 | -11743 | -10.1 |
| 2021 | 55-64 & F | 159219 | -6068 | -3.81 |
| 2021 | 55-64 & M | 157169 | -10632 | -6.76 |
| 2021 | ≥65 & F | 342167 | -14723 | -4.30 |
| 2021 | ≥65 & M | 324098 | -13632 | -4.21 |

**Table S3** Real ED Visit absolute counts in same-period 2019 and percentage changes of each wave/after-wave period, across various age and gender groups.

| Groups | Wave Period | 2016-2019 average | Change | Change (%) |
| --- | --- | --- | --- | --- |
| 0-17 & F | 1 | 9132 | -1444 | -15.8 |
| 0-17 & F | 2 | 14526 | -10345 | -71.2 |
| 0-17 & F | 3 | 18415 | -13448 | -73 |
| 0-17 & F | 4 | 14576 | -9198 | -63.1 |
| 0-17 & F | after-wave | 35421 | -22597 | -63.8 |
| 0-17 & M | 1 | 171651 | -85823 | -50 |
| 0-17 & M | 2 | 11755 | -1619 | -13.8 |
| 0-17 & M | 3 | 18329 | -12823 | -70 |
| 0-17 & M | 4 | 24185 | -17715 | -73.2 |
| 0-17 & M | after-wave | 18848 | -11851 | -62.9 |
| 18-34 & F | 1 | 45832 | -29415 | -64.2 |
| 18-34 & F | 2 | 225470 | -115802 | -51.4 |
| 18-34 & F | 3 | 12371 | -190 | -1.54 |
| 18-34 & F | 4 | 19170 | -6663 | -34.8 |
| 18-34 & F | after-wave | 26300 | -9193 | -35 |
| 18-34 & M | 1 | 25326 | -6557 | -25.9 |
| 18-34 & M | 2 | 47776 | -17089 | -35.8 |
| 18-34 & M | 3 | 240055 | -50554 | -21.1 |
| 18-34 & M | 4 | 10702 | 238 | 2.22 |
| 18-34 & M | after-wave | 16396 | -2874 | -17.5 |
| 35-44 & F | 1 | 22304 | -4857 | -21.8 |
| 35-44 & F | 2 | 23027 | -2197 | -9.54 |
| 35-44 & F | 3 | 41690 | -11044 | -26.5 |
| 35-44 & F | 4 | 209609 | -28681 | -13.7 |
| 35-44 & F | after-wave | 8520 | -710 | -8.33 |
| 35-44 & M | 1 | 12986 | -4472 | -34.4 |
| 35-44 & M | 2 | 18775 | -6780 | -36.1 |
| 35-44 & M | 3 | 17674 | -4617 | -26.1 |
| 35-44 & M | 4 | 33245 | -11126 | -33.5 |
| 35-44 & M | after-wave | 171095 | -28117 | -16.4 |
| 45-54 & F | 1 | 6525 | -207 | -3.17 |
| 45-54 & F | 2 | 9806 | -1957 | -20 |
| 45-54 & F | 3 | 13834 | -2995 | -21.6 |
| 45-54 & F | 4 | 14277 | -1530 | -10.7 |
| 45-54 & F | after-wave | 25602 | -5423 | -21.2 |
| 45-54 & M | 1 | 131922 | -13898 | -10.5 |
| 45-54 & M | 2 | 10023 | -770 | -7.68 |
| 45-54 & M | 3 | 14898 | -5484 | -36.8 |
| 45-54 & M | 4 | 22058 | -8301 | -37.6 |
| 45-54 & M | after-wave | 21626 | -5464 | -25.3 |
| 55-64 & F | 1 | 39370 | -11103 | -28.2 |
| 55-64 & F | 2 | 203543 | -20187 | -9.92 |
| 55-64 & F | 3 | 7509 | -608 | -8.1 |
| 55-64 & F | 4 | 11312 | -3379 | -29.9 |
| 55-64 & F | after-wave | 16068 | -4742 | -29.5 |
| 55-64 & M | 1 | 16582 | -2986 | -18 |
| 55-64 & M | 2 | 29487 | -6506 | -22.1 |
| 55-64 & M | 3 | 151606 | -15162 | -10 |
| 55-64 & M | 4 | 10216 | 44 | 0.431 |
| 55-64 & M | after-wave | 15394 | -5931 | -38.5 |
| ≥65 & F | 1 | 22058 | -7820 | -35.5 |
| ≥65 & F | 2 | 22034 | -5678 | -25.8 |
| ≥65 & F | 3 | 41106 | -9440 | -23 |
| ≥65 & F | 4 | 206670 | -11036 | -5.34 |
| ≥65 & F | after-wave | 9925 | -35 | -0.353 |
| ≥65 & M | 1 | 15288 | -4535 | -29.7 |
| ≥65 & M | 2 | 21544 | -5732 | -26.6 |
| ≥65 & M | 3 | 22383 | -3724 | -16.6 |
| ≥65 & M | 4 | 40098 | -7815 | -19.5 |
| ≥65 & M | after-wave | 204208 | -13875 | -6.79 |

**Table S4** Estimated Excess 28d Mortality per 100 reduced ED Visits across ≥65 male and female groups, and wave periods, with time lag up to 14 days.

| Gender | Time Lag (Days) | Mean Estimate | Lower 95CI | Higher 95CI |
| --- | --- | --- | --- | --- |
| F | 0 | 1.15 | 0.763 | 1.54 |
| F | 1 | 1.16 | 0.787 | 1.54 |
| F | 2 | 1.14 | 0.759 | 1.51 |
| F | 3 | 1.13 | 0.755 | 1.51 |
| F | 4 | 1.12 | 0.745 | 1.5 |
| F | 5 | 1.07 | 0.694 | 1.45 |
| F | 6 | 1.08 | 0.699 | 1.45 |
| F | 7 | 1.04 | 0.658 | 1.42 |
| F | 8 | 0.998 | 0.624 | 1.37 |
| F | 9 | 0.95 | 0.575 | 1.32 |
| F | 10 | 0.972 | 0.594 | 1.35 |
| F | 11 | 0.941 | 0.564 | 1.32 |
| F | 12 | 0.895 | 0.521 | 1.27 |
| F | 13 | 0.896 | 0.521 | 1.27 |
| F | 14 | 0.883 | 0.504 | 1.26 |
| M | 0 | 0.953 | 0.382 | 1.52 |
| M | 1 | 0.972 | 0.412 | 1.53 |
| M | 2 | 0.898 | 0.34 | 1.45 |
| M | 3 | 0.928 | 0.372 | 1.48 |
| M | 4 | 0.956 | 0.398 | 1.51 |
| M | 5 | 0.921 | 0.366 | 1.48 |
| M | 6 | 0.839 | 0.284 | 1.39 |
| M | 7 | 0.708 | 0.153 | 1.26 |
| M | 8 | 0.699 | 0.146 | 1.25 |
| M | 9 | 0.714 | 0.161 | 1.27 |
| M | 10 | 0.726 | 0.174 | 1.28 |
| M | 11 | 0.741 | 0.189 | 1.29 |
| M | 12 | 0.773 | 0.22 | 1.33 |
| M | 13 | 0.752 | 0.198 | 1.31 |
| M | 14 | 0.742 | 0.188 | 1.3 |

**Table S5** Estimated ≥65 male and female Excess 28d Mortality by Reduced ED Visit with time lag up to 14 days.

| Gender | Time Lag  (Days) | 2020 |  |  | 2021 |  |  |
| --- | --- | --- | --- | --- | --- | --- | --- |
|  |  | **Mean Estimate** | **Lower 95 CI** | **Higher 95CI** | **Mean Estimate** | **Lower 95CI** | **Higher 95CI** |
| F | 0 | 969 | 642 | 1300 | 237 | 157 | 317 |
| F | 1 | 980 | 662 | 1300 | 240 | 162 | 317 |
| F | 2 | 956 | 639 | 1270 | 234 | 156 | 311 |
| F | 3 | 955 | 636 | 1270 | 234 | 155 | 312 |
| F | 4 | 945 | 627 | 1260 | 231 | 153 | 309 |
| F | 5 | 901 | 584 | 1220 | 220 | 143 | 298 |
| F | 6 | 905 | 589 | 1220 | 221 | 144 | 299 |
| F | 7 | 872 | 554 | 1190 | 213 | 135 | 291 |
| F | 8 | 840 | 525 | 1160 | 206 | 129 | 283 |
| F | 9 | 800 | 484 | 1110 | 196 | 118 | 273 |
| F | 10 | 818 | 500 | 1140 | 200 | 122 | 278 |
| F | 11 | 792 | 475 | 1110 | 194 | 116 | 271 |
| F | 12 | 753 | 438 | 1070 | 184 | 107 | 261 |
| F | 13 | 754 | 439 | 1070 | 184 | 107 | 262 |
| F | 14 | 743 | 424 | 1060 | 182 | 104 | 260 |
| M | 0 | 590 | 236 | 944 | 162 | 64.9 | 259 |
| M | 1 | 602 | 256 | 949 | 165 | 70.2 | 261 |
| M | 2 | 556 | 211 | 901 | 153 | 57.9 | 248 |
| M | 3 | 575 | 230 | 920 | 158 | 63.2 | 253 |
| M | 4 | 592 | 247 | 938 | 163 | 67.7 | 257 |
| M | 5 | 571 | 227 | 914 | 157 | 62.3 | 251 |
| M | 6 | 520 | 176 | 864 | 143 | 48.4 | 237 |
| M | 7 | 439 | 94.5 | 783 | 120 | 26 | 215 |
| M | 8 | 433 | 90.6 | 775 | 119 | 24.9 | 213 |
| M | 9 | 442 | 99.8 | 784 | 121 | 27.4 | 215 |
| M | 10 | 450 | 108 | 792 | 123 | 29.6 | 217 |
| M | 11 | 459 | 117 | 801 | 126 | 32.1 | 220 |
| M | 12 | 479 | 136 | 821 | 132 | 37.5 | 226 |
| M | 13 | 466 | 123 | 809 | 128 | 33.7 | 222 |
| M | 14 | 460 | 116 | 803 | 126 | 32 | 221 |

**Table S6** ≥65 28d mortality change between 2016-2019 average, and 2020 and 2021 respectively, by DOA/DBA and LTC residence status.

| Year | DOA/DBA | LTC | 2016-2019 average | Change | Change (%) |
| --- | --- | --- | --- | --- | --- |
| 2020 | N | N | 15444 | 678 | 4.39 |
| 2020 | N | Y | 8760 | 524 | 5.98 |
| 2020 | Y | N | 3026 | 1284 | 42.4 |
| 2020 | Y | Y | 1310 | 173 | 13.2 |
| 2021 | N | N | 15444 | 1366 | 8.84 |
| 2021 | N | Y | 8760 | 806 | 9.20 |
| 2021 | Y | N | 3026 | 1055 | 34.9 |
| 2021 | Y | Y | 1310 | 94 | 7.18 |

LTC: Long-term care, Y for residents N for non-residents

**Table S7** ≥65 Visit changes between 2016-2019 average and 2020-2021, across LTC status and periods.

| LTC | Wave | 2016-2019 average | Change | Change (%) |
| --- | --- | --- | --- | --- |
| N | 1 | 58068 | -20919 | -36 |
| N | 2 | 76825 | -24053 | -31.3 |
| N | 3 | 75996 | -19535 | -25.7 |
| N | 4 | 152990 | -29389 | -19.2 |
| N | after-wave | 724082 | -42575 | -5.88 |
| Y | 1 | 11155 | -1998 | -17.9 |
| Y | 2 | 13929 | -2293 | -16.5 |
| Y | 3 | 13817 | -1873 | -13.6 |
| Y | 4 | 28189 | -1792 | -6.36 |
| Y | after-wave | 129464 | -2102 | -1.62 |

LTC: Long-term care, Y for residents N for non-residents

**Table S8** Breakdown of individual elderly DOA/DBA by COVID group, visit rate changes and LTC status.

|  | **COVID group*** |  | **Pre-COVID group*** |  |
| --- | --- | --- | --- | --- |
|  | **Non-LTC** | **LTC** | **Non-LTC** | **LTC** |
| Increase in visit rate | 92 | 102 | 100 | 122 |
| No Visit in Treatment Period | 280 | 147 | 112 | 89 |
| Reduction in visit rate | 171 | 136 | 122 | 113 |

Pearson's Chi-square test p-value < 0.05.

**Table S9** Breakdown of individual elderly DOA/DBA by COVID group, visit rate changes and LTC status.

|  | **Non-LTC*** |  | **LTC**** |  |
| --- | --- | --- | --- | --- |
|  | **COVID group** | **Pre-COVID group** | **COVID group** | **Pre-COVID group** |
| Increase in visit rate | 92 | 100 | 102 | 122 |
| No Visit in Treatment Period | 280 | 112 | 147 | 89 |
| Reduction in visit rate | 171 | 122 | 136 | 113 |

Pearson’s Chi-square test p-value < 0.05; Pearson’s Chi-square test p-value = 0.08

**[not cited in MS]** Scatter plots of Episode Admission and 28-day All-Cause Mortality Yearly Percentage Change of ≥65 comorbidity plots, during waves and non-wave period in 2021, with Absolute Seasonally-Adjusted Change of 28d Mortality in parentheses. The subgroup excludes DOA/DBA. Only comorbidities with deaths in corresponding period over 10 are included. Blue line is 1:1 visit and death change line, high mortality-rate situations like cardiac arrest and sudden death normally fall on this line.

*
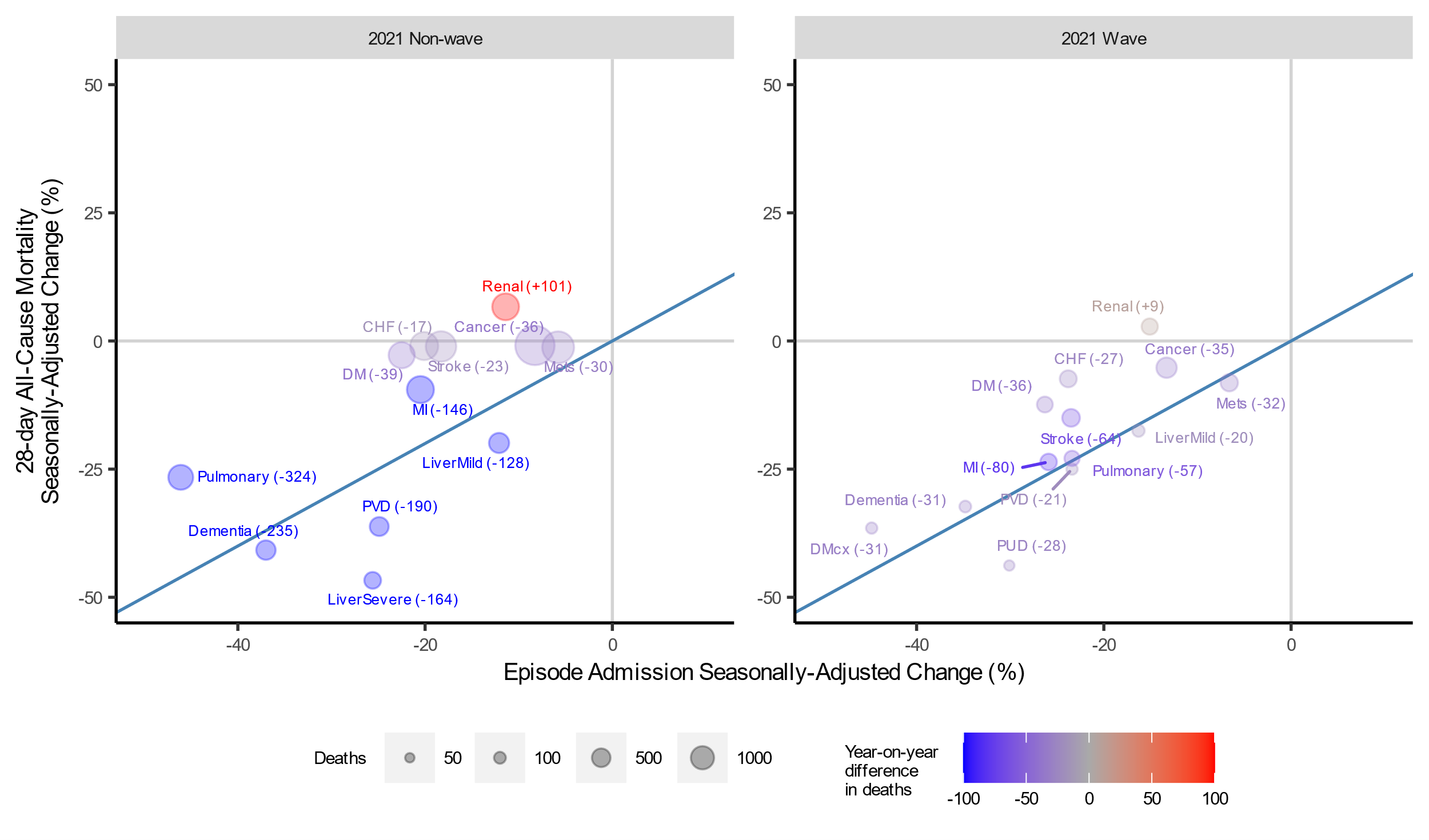
*

**References:**

1 Hospital Authority. Enhanced Laboratory Surveillance. https://www.ha.org.hk/visitor/ha_visitor_index.asp?Content_ID=255014 (accessed May 17, 2021).

2 Centre for Health Protection, HKSAR Government. CHP investigates highly suspected imported case of novel coronavirus infection. https://www.info.gov.hk/gia/general/202001/22/P2020012200982.htm?fontSize=1 (accessed May 17, 2021).

3 HKSAR Government. Gradual resumption of services. 2020; published online Feb 27. https://www.news.gov.hk/eng/2020/02/20200227/20200227_215410_929.html.

4 HKSAR Government. World travel alert to be raised. 2020; published online March 17. https://www.news.gov.hk/eng/2020/03/20200317/20200317_110525_721.html.

5 HKSAR Government. CE explains relaxation of measures. 2020; published online May 5. https://www.news.gov.hk/eng/2020/05/20200505/20200505_171110_984.html.

6 HKSAR Government. Social distancing rules to be tightened. 2020; published online July 9. https://www.news.gov.hk/eng/2020/07/20200709/20200709_175812_722.html.

7 HKSAR Government. Anti-epidemic measures to be relaxed. 2020; published online Aug 25. https://www.news.gov.hk/eng/2020/08/20200825/20200825_141754_497.html.

8 HKSAR Government. Social distancing measures tightened. 2020; published online Nov 14. https://www.news.gov.hk/eng/2020/11/20201114/20201114_181615_268.html.

9 HKSAR Government. Govt may ease social distancing. 2021; published online Feb 10. https://www.news.gov.hk/eng/2021/02/20210210/20210210_184511_308.html?type=category&name=health&tl=t.
